# Urachal carcinoma: A novel staging system utilizing the National Cancer Database

**DOI:** 10.1002/cam4.5164

**Published:** 2022-09-07

**Authors:** Vladimir Limonnik, Arash Samiei, Stephen Abel, Rodney E. Wegner, Goutham Vemana, Shifeng S. Mao

**Affiliations:** ^1^ Department of Internal Medicine Allegheny Health Network Pittsburgh Pennsylvania USA; ^2^ Division of Urology Allegheny Health Network Pittsburgh Pennsylvania USA; ^3^ Division of Radiation Oncology Allegheny Health Network Cancer Institute Pittsburgh Pennsylvania USA; ^4^ Division of Medical Oncology Allegheny Health Network Cancer Institute Pittsburgh Pennsylvania USA; ^5^ Department of Hematology/Oncology Lankenau Medical Center Wynnewood Pennsylvania USA

**Keywords:** cancer, chemotherapy, genitourinary cancer, malignancy, Mayo staging, National Cancer Database, Ontario staging, Sheldon staging, staging systems, TNM, Urachal carcinoma, urachus, urinary bladder

## Abstract

**Background:**

Urachal carcinoma (UrC) is a rare, aggressive cancer with a poor prognosis that is frequently diagnosed in advanced stages. Due to its rarity, the current staging systems, namely Sheldon, Mayo, and Ontario were established based on relatively small patient cohorts, necessitating further validation. We used a large patient population from the National Cancer Database to model a novel staging system based on the Tumor (T), Node(N), and Metastasis (M) (TNM) staging system and compared it to established staging systems.

**Methods:**

We identified patients diagnosed with UrC between the years of 2004–2016. To determine median overall survival (OS), a Kaplan–Meier (KM) curve was generated using the Sheldon, Mayo, Ontario, and TNM staging system. A cox proportional‐hazards regression model was developed to highlight predictors of overall survival.

**Results:**

A total of 626 patients were included in the analysis. The OS for the entire cohort was 58.2 months (50.1–67.8) with survival rates at 12, 24, and 60 months of 83%, 70%, and 49%, respectively (*p* < 0.0001). As compared to the Sheldon, Mayo, and Ontario staging system, our TNM staging system had a more balanced sample and survival distribution per stage and no overlap among stages on KM survival curves. The Mayo, Ontario, and TNM staging systems were more accurate in terms of stage‐survival correlation than the Sheldon staging system (*p* < 0.05 for all stages).

**Conclusions:**

The proposed novel TNM staging system for UrC has a more balanced sample distribution and a more accurate stage‐survival correlation than the traditional Mayo, Sheldon, and Ontario staging systems. It is clinically applicable and enables better risk stratification, prognosis, and therapeutic decision‐making.

## Graphical abstract

This study proposed a novel TNM staging system for urachal carcinoma based a large number of patients in NCDB. It provides a more balanced sample distribution and a more accurate stage‐survival correlation than the traditional Sheldon, Mayo, and Ontario staging systems. It is clinically applicable and enables better risk stratification, prognosis, and therapeutic decision‐making.

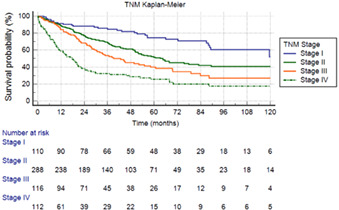

## 1 INTRODUCTION

The urachus, a fibrous remnant of the allantois, is a vestigial structure that connects the urinary bladder to the allantois during the early stages of embryonic development. The fibrous remnant persists after birth as the median umbilical ligament. Urachal tissue remnants that remain in the ligament have the potential to become neoplastic at any point along the tract.^1^ Urachal carcinoma (UrC) is a rare, highly malignant epithelial cancer that commonly arises at the dome or anterior wall of the bladder. Several histologic subtypes of UrC exist; adenocarcinoma is the most common subtype.^2^, ^3^, ^4^, ^5^ Given that tumors typically infiltrate the muscularis mucosa and urothelial surface, hematuria is the most common clinical presentation.^6^ As tumors are frequently diagnosed at advanced stages, the prognosis remains poor with 5‐year overall survival rates around 50%.^7^ There are currently no definitive treatment guidelines for UrC due to the rarity of the cancer and the lack of large, randomized, prospective studies. Rather, treatment is extrapolated from small‐series case reports. Furthermore, several staging systems for UrC have been proposed, which have not been validated.^8^ There is no American Joint Committee on Cancer (AJCC) TNM staging system for UrC. Although the urachal remnant is commonly lined with urothelium that is similar to that of the bladder, UrC does not originate from the bladder surface urothelium and thus exhibits biological, clinical, and pathologic characteristics distinct from those of bladder cancers.^9^ As a result, the urinary bladder TNM staging system's applicability to UrC is limited. However, Dhillon et al. and Molina et al. demonstrated that TNM staging can be applied to UrC and has prognostic utility.^9^, ^10^ Sheldon et al. proposed the first staging system in 1984, known as the Sheldon staging system. Subsequently, Ashley et al. and Pinthus et al. developed alternate systems in 2006, known as the Mayo and Ontario staging systems, respectively^4^, ^6^, ^11^ (Table 1). These three staging systems are commonly used but have many limitations, including limited sample size, and require further validation.

**TABLE 1:** Comparison of different staging systems for Urachal carcinoma

Proposed TNM	Sheldon^4^	Mayo^6^	Ontario^11^
I: confined to the urachus submucosa	I: no invasion beyond urachal mucosa	I: confined to the urachus and/or bladder	T1: confined to the submucosa
II: invasion of bladder muscularis propria or microscopic invasion of bladder perivesical tissue	II: invasion confined to urachus	II: extending beyond the muscular layer of the urachus and/or bladder	T2: confined to the muscular wall of the bladder
III: macroscopic invasion of bladder perivesical tissue or invasion of nearby structures, including uterus/vagina/prostate	III: local extension into A: bladder B: abdominal wall C: peritoneum D: viscera other than bladder	III: infiltrating the regional lymph nodes	T3: extends into the peri‐urachal or vesical soft tissue
IV: invasion of the pelvic/abdominal wall/peritoneum or any nodal or distant site involvement	IV: metastasis to A: regional lymph nodes B: distant sites	IV: infiltrating non‐regional lymph nodes or other distant sites	T4: invades adjacent organs, including the abdominal wall

Given the lack of consensus regarding the ideal staging system for UrC, we queried the National Cancer Database (NCDB) in order to compare established staging systems, namely Sheldon, Mayo, and Ontario against our novel, modified Tumor, Nodes, Metastases (TNM) staging system.

## 2 METHODS

### 2.1 Patient selection

**FIGURE 1:**
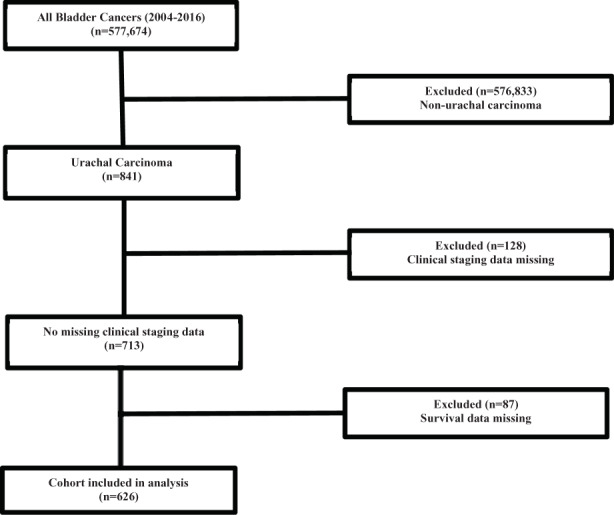
CONSORT diagram Urachal carcinoma

We conducted a retrospective hospital‐based review using de‐identified patient data from the NCDB, a large clinical oncology database that captures more than 70% of newly diagnosed malignancies in the United States and which is supported by the American Cancer Society, American College of Surgeons (ACS), and Commission on Cancer (CoC).^12^ Due to the de‐identification of patient data, the study was exempt from institutional review board supervision. The NCDB Participant User File (PUF) was used to identify patients diagnosed with UrC between 2004 and 2016 (ICD‐O‐3 code C67.7). Patients with non‐urachal carcinoma, missing clinical staging data, and missing survival data were excluded from the study. The CONSORT diagram depicted in Figure 1 outlines the study cohort selection process. UrC was identified in a total of 841 patients of which 626 patients met the study's inclusion criteria and were included in the analysis; however, only 451 patients were eligible for the Ontario system analysis because it did not include patients with nodal or distant metastasis as part of the staging.

### 2.2 Staging

Several staging systems have been proposed for UrC, including those by Sheldon et al., Ashley et al., and Pinthus et al.^4^, ^6^, ^11^ (Table 1). Based on their experience with five patients with UrC, Sheldon et al. defined a four‐stage system in 1984.^4^ The Sheldon staging system was defined as follows: Stage I, no invasion beyond the urachal mucosa; stage II, invasion confined to the urachus; stage IIIA, IIIB, IIIC, IIID, local extension into the bladder, abdominal wall, peritoneum, or viscera other than the bladder, respectively; stage IV‐A, IV‐B, metastatic to the regional lymph nodes or metastatic to distant sites, respectively. In 2006, Ashley et al. described a novel four‐stage system, known as the Mayo staging system, based on a larger cohort of 66 patients,^6^ as follows: stage I, tumors confined to the urachus and/or bladder; stage II, tumors extending beyond the muscular layer of the urachus and/or the bladder; stage III, tumors infiltrating the regional lymph nodes; stage IV, tumors infiltrating nonregional lymph nodes or other distant sites. Additionally, Pinthus et al. described a four‐stage system, known as the Ontario staging system, based on 32 patients treated operatively, as follows: T1, tumor confined to the submucosa; T2, tumor confined to the muscular wall of the bladder; T3, tumor extends into the peri‐urachal or vesical soft tissue; T4, tumor invades adjacent organs, including the abdominal wall; notably, nodal stage and metastatic status was not available and not included in the staging system.^11^

Our novel, four‐category TNM staging system is described in Tables 1 and 2. Stage 1 disease (T‐stage 0, a, *in‐situ* (Tis), 1; absence of nodal involvement or distant metastasis) is confined to the urachus. Stage 2 disease (T‐stage 2, 3, 3a; absence of nodal involvement or distant metastasis) involves tumor invasion of the bladder muscularis propria or microscopic invasion of bladder perivesical tissue. Stage 3 disease (T‐stage 3b, 4a; absence of nodal involvement or distant metastasis) involves macroscopic invasion of bladder perivesical tissue or invasion of nearby structures, including the uterus, vagina, or prostate. Stage 4 disease (T‐stage 4b with absence of nodal involvement or distant metastasis; any T stage with nodal involvement but lack of distant metastasis; any T stage and any N stage with distant metastasis) involves tumor invasion of the pelvic/abdominal wall or any nodal or distant site involvement.

**TABLE 2:** Proposed TNM staging system for Urachal carcinoma

Stage	Primary tumor (T)	Regional lymph nodes (N)	Distant metastasis (M)
Stage I	T0, Ta, Tis, T1	N0	M0
Stage II	T2, T3, T3a	N0	M0
Stage III	T3b, T4a	N0	M0
Stage IV	T4b	N0	M0
	Tx	N1–N3	M0/M1
	Any T	N1–N3	M0
	Any T	Any N	M1

Although an AJCC TNM staging system for the urinary bladder exists, currently in its 8th edition,^13^ it cannot be directly applied to UrC given that the urachus develops from urachal remnants that are mainly found in the bladder wall or extravesical midline.^5^ However, due to NCDB's inherent limitations, UrC cases in the dataset were coded using the 6th or 7th edition of the *AJCC Cancer Staging Manual* for the urinary bladder.^14^, ^15^ In order to perform a comparative analysis between the Sheldon, Mayo, Ontario, and our novel, four‐category TNM staging system, we first sought to define the AJCC TNM case coding used in the NCDB as it relates to the Mayo, Sheldon, and Ontario staging systems. To summarize, primary tumor (T) stages 0, a, *in‐situ*, 1, 2, 2a, and 2b were defined as Mayo stage 1 because the tumor is confined to the urachus and/or bladder muscularis propria. T‐stages 3, 3a, 3b, 4, 4a, 4b correlated with Mayo stage 2 due to tumor extension beyond the urachus/bladder muscular layer and with invasion of bladder perivesical tissue or nearby structures including the pelvic/abdominal wall, uterus, or vagina. Any regional lymph node (N) involvement, N1‐N3, was defined as Mayo stage 3 and any distant metastasis (M) was defined as Mayo stage 4. Sheldon stage 1 corresponded with T‐stage 0, a, and *in‐situ* given no tumor invasion beyond the urachal mucosa. Sheldon stage 2 equated with T‐stage 1 as tumor invasion is still confined to the urachus. Sheldon stages 3A/B/C/D were grouped and corresponded with T‐stages 2, 2a, 2b, 3, 3a, 3b, 4, 4a, and 4b due to tumor invasion of perivesical tissue or nearby structures such as the bladder, abdominal/pelvic wall, uterus, vagina, or other viscera. Any regional lymph node involvement, N1‐N3, was defined as Sheldon 4A and any distant metastasis, M1, corresponded with Sheldon 4B. For the Ontario system, T1 included T‐stages 0, a, *in‐situ*, and 1 as the tumor is confined to the urachal submucosa. T2 correlated with T‐stage 2 as the tumor is limited to the muscular wall of the bladder. T3 corresponded to T‐stages 3, 3a, 3b as the tumor invades perivesical tissue and T4 correlated to T‐stage 4a and 4b given invasion of adjacent organs.

### 2.3 Statistics

Data analysis was performed utilizing MedCalc Version 19.6. Kaplan–Meier (KM) curves were created to determine the cumulative probability of survival.^16^ Overall survival (OS), in months, was determined by calculating the difference in time between initial diagnosis date and date of death or last contact. For multivariable survival analysis, a Cox proportional‐hazards regression model was developed and used.^16^ Notably, neither the ACS nor the CoC validated the statistical methods utilized in this study, and thus are not responsible for the conclusions reached by the researchers based on these data.

## 3 RESULTS

### 3.1 Patient characteristics

The cohort's baseline characteristics included mostly patients who were white (81%), male (56%), and had a median age of 58 years. The stage distribution of UrC cases according to Sheldon, Mayo, Ontario, and TNM staging criteria were as follows: Sheldon stage I (7%), stage II (9%), stage III (56%), stage IV (28%); Mayo stage I (33%), stage II (38%), stage III (7%), stage IV (21%); Ontario T1 (22%), T2 (24%), T3 (45%), T4 (9%); TNM stage I (18%), stage II (46%), stage III (18%), stage IV (18%) (Table 3). The cohort had an overall Charlson/Deyo comorbidity score of 0 (80%).^17^

**TABLE 3:** Survival proportions for Urachal carcinoma based on Sheldon, Mayo, Ontario, and TNM staging systems

Staging system	Stage distribution		Median OS (months), (95% CI)	Survival proportion		
	Number	Percentage		12‐months	24‐months	60‐months
Sheldon						
Stage I	43	7	132.2 (89.2–132.2)	0.90	0.85	0.85
Stage II	57	9	Undefined (Undefined)	0.94	0.94	0.74
Stage III	349	56	69.6 (60.8–89.5)	0.91	0.82	0.57
Stage IV	177	28	17.3 (14.2–20.7)	0.62	0.37	0.17
Mayo						
Stage I	209	33	132.2 (88.6–132.2)	0.91	0.85	0.67
Stage II	240	38	69.5 (58.5–88.4)	0.91	0.82	0.57
Stage III	46	7	19.7 (11.6–32.0)	0.65	0.41	0.17
Stage IV	131	21	16.8 (13.2–20.7)	0.61	0.37	0.17
Ontario						
T1	100	22	132.2 (89.2–132.2)	0.92	0.90	0.78
T2	110	24	77.9 (51.8–77.9)	0.90	0.80	0.57
T3	201	45	75.5 (58.2–89.5)	0.93	0.86	0.56
T4	40	9	68.0 (21.1–88.2)	0.81	0.64	0.57
TNM						
Stage I	110	18	132.2 (88.6–132.2)	0.91	0.88	0.74
Stage II	288	46	64.6 (51.8–83.0)	0.89	0.77	0.52
Stage III	116	18	42.7 (30.1–58.2)	0.84	0.68	0.40
Stage IV	112	18	17.8 (11.0–21.1)	0.59	0.38	0.25
Overall	626	100	58.2 (50.1–67.8)	0.83	0.70	0.49

Abbreviation: CI, confidence interval.

### 3.2 Survival

**FIGURE 2:**
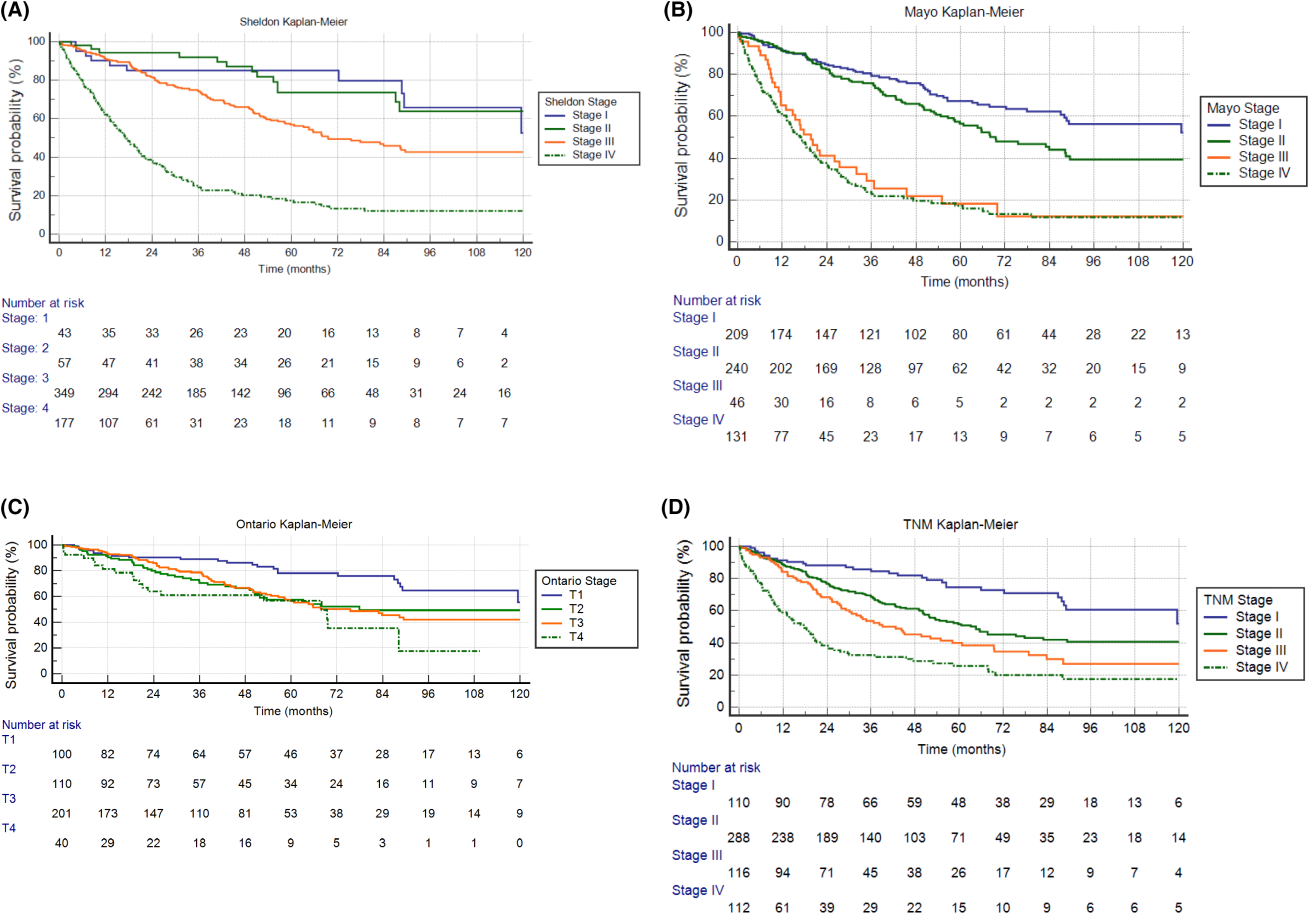
KM curve for Urachal carcinoma based on (A) Sheldon staging; (B) Mayo staging; (C) Ontario staging; (D) proposed TNM staging system. KM, Kaplan–Meier

To determine median OS for UrC, a KM curve was generated using the Sheldon, Mayo, Ontario, and TNM staging systems. The OS for the entire cohort of 626 patients was 58.2 months (95% Confidence Interval [CI]: 50.1–67.8) with survival rates at 12, 24, and 60 months being 83%, 70%, and 49%, respectively (*p* < 0.0001) (Table 3). Based on the Sheldon staging system (Figure 2A), the median OS was 132.2 (95% CI: 89.2–132.2) for stage 1, undefined due to survival curve not crossing 50% survival for stage 2, 69.6 (95% CI: 60.8–89.5) for stage 3, and 17.3 (95% CI: 14.2–20.7) months for stage 4 (*p* < 0.0001 for all stages). According to the Mayo staging system (Figure 2B), the median OS was 132.2 (95% CI: 88.6–132.2), 69.5 (95% CI: 58.5–88.4), 19.7 (95% CI: 11.6–32.0), and 16.8 (95% CI: 13.2–20.7) months for stage 1, 2, 3, and 4, respectively (*p* < 0.0001). Per the Ontario staging system (Figure 2C), the median OS was 132.2 (95% CI: 89.2–132.2), 77.9 (95% CI: 51.8–77.9), 75.5 (95% CI: 58.2–89.5), and 68.0 (95% CI: 21.1–88.2) months for T1, 2, 3, and 4, respectively (*p* < 0.0001). The median OS based on the proposed TNM staging system (Figure 2D) was 132.2 (95% CI: 88.6–132.2), 64.6 (95% CI: 51.8–83.0), 42.7 (95% CI: 30.1–58.2), and 17.8 (95% CI: 11.0–21.1) months for stage 1, 2, 3, and 4, respectively (*p* < 0.0001). Table 3 depicts median OS, in months, along with 12, 24, and 60‐month survival proportions for each staging system. When compared to the Mayo, Sheldon, and Ontario staging systems, the proposed TNM staging system has a more balanced sample distribution per stage.

A cox proportional‐hazards regression model was developed to identify predictors of overall survival (Table 4). The Mayo, Ontario, and our proposed TNM staging system demonstrated more accurate stage‐survival correlation (*p* < 0.05 for all stages) than the Sheldon staging system.

**TABLE 4:** Cox proportional‐hazards regression model for overall survival using the Sheldon, Mayo, Ontario, and TNM staging systems

Staging system	Hazard of death	95% CI	*p*‐value
Sheldon			
Stage I	Reference	Reference	Reference
Stage II	0.89	0.40–1.98	0.77
Stage III	1.82	0.98–3.36	0.06
Stage IV	6.60	3.56–12.23	**<0.0001**
Mayo			
Stage I	Reference	Reference	Reference
Stage II	1.40	1.02–1.92	**0.0390**
Stage III	4.40	2.90–6.70	**<0.0001**
Stage IV	5.19	3.80–7.10	**<0.0001**
Ontario			
T1	Reference	Reference	Reference
T2	1.93	1.17–3.21	**0.010**
T3	1.93	1.22–3.06	**0.005**
T4	3.01	1.64–5.51	**0.0004**
TNM			
Stage I	Reference	Reference	Reference
Stage II	1.87	1.25–2.79	**0.0022**
Stage III	2.74	1.78–4.22	**<0.0001**
Stage IV	4.96	3.26–7.56	**<0.0001**

*Note*: Values in bold are statistically significant (*p* < 0.05).

Abbreviation: CI, confidence interval; TNM, tumor, node and metastasis.

## 4 DISCUSSION

UrC is a rare malignancy accounting for <1% of all bladder malignancies.^2^, ^3^ Several staging systems for UrC have been proposed however have not been validated. Furthermore, evidence‐based treatment guidelines are not well established given the lack of large, randomized, prospective studies. Thus, treatment of UrC is extrapolated from small‐series case reports and single‐institution studies.^8^ There remains a crucial need for a validated staging system in order to help inform clinicians and patients regarding prognosis and guide treatment decisions.

Our study represents one of the largest known analysis of UrC patients. We found that our proposed TNM staging system may serve as a novel staging system for UrC and aid in disease prognostication and management; however, our staging system requires further validation and comparison against established staging systems.

Multiple staging systems for UrC have been proposed; the Sheldon and Mayo staging systems are the most commonly used.^4^, ^6^, ^7^, ^10^ In 1984, Sheldon et al. proposed the first staging system based on their experience with five patients, all of whom had advanced disease, either Sheldon stage III or IV, and underwent cystectomy.^4^ Ashley et al. developed the Mayo staging system in 2006 based on 66 patients that were evaluated at the Mayo Clinic from 1951 to 2004. The majority of patients were Mayo stage I and II, 42% and 29%, respectively.^6^ In the same year, Pinthus et al. developed the Ontario staging system based on a cohort of 40 patients identified in the Ontario Cancer Registry from 1976 to 2001; among them, 32 patients were treated operatively. Only T‐stage was defined in the Ontario system; the majority of cases, 59.4%, were stage T3.^11^

Szarvas et al. conducted a meta‐analysis of 1010 UrC cases using single‐institution and population‐based studies; of those patients, 532 had data available for Sheldon staging and 221 were staged according to the Mayo system. It was found that the majority of cases were staged as Sheldon stage III and IV, 68% and 24%, respectively, and Mayo stage I and II, 33% and 40%, respectively. Sheldon staging tends to classify patients as higher stage disease, with nearly 92% of UrC diagnosed at advanced stages (Sheldon stage III and greater) and at which point there is already tumor invasion of the urinary bladder. Mayo staging classifies patients as lower stage and seems to have a more balanced patient distribution by stage.^7^ Similarly, Dhillon et al. conducted a retrospective analysis of 46 UrC cases and found that all of the cases were staged as either Sheldon stage III or IV, 59% and 41%, respectively, supporting the notion that Sheldon staging tends to classify patients as higher stage disease.^9^ Likewise, Pinthus et al. performed a retrospective analysis of 32 UrC patients treated operatively, noting only one patient correlated with Sheldon stage I disease, no patients with stage II disease, and the majority of cases, 68.8%, had stage III disease.^11^ Interestingly, Hamilou et al. compared the Sheldon and Mayo systems and demonstrated that both systems were capable of predicting cancer‐specific mortality.^8^ Our analysis corroborated the uneven distribution of staging by the Sheldon, Mayo, and Ontario systems, demonstrating that the majority of cases were Sheldon stages III and IV, 56% and 28%, respectively, Mayo stages I and II, 33% and 38%, respectively, and Ontario stage T3, 45%. Multiple studies have described that the stage of disease is one of the most important prognostic factors in UrC.^18^, ^19^ Given that prognosis and treatment selection are dependent on UrC staging, our TNM system exhibits a more balanced distribution of stages, which may assist in risk stratification and guide therapeutic decisions.

Although an AJCC TNM staging system exists for the urinary bladder, it cannot be applied directly to UrC.^5^, ^9^ Interestingly, several authors have shown that TNM staging may be applied to UrC and has prognostic utility. In a study by Molina et al., clinical outcomes of 49 patients with UrC were analyzed. They found overall survival among all stages to be 62 months, similar to our analysis which found overall survival to be 58 months. Using the TNM staging system, they found that median survival time for stage I and II was greater than 10 years and 7.5 years, respectively, and drastically decreased with stage III and IV disease, 1–2 years and <1 year, respectively.^10^ Similarly, utilizing our novel TNM staging system, we found a median overall survival of 11 years and greater than 5 years in stage I and II disease, respectively, and a considerable decrease in survival time with stage III and IV disease, 3.5 years and <1.5 years, respectively. Furthermore, Dhillon et al. evaluated the TNM staging system by adapting the AJCC TNM staging system for the urinary bladder.^15^ Importantly, they found that the TNM staging system has prognostic utility when applied to UrC. They showed that cancer‐specific survival was associated with the TNM stage, as patients with higher stage disease had considerably higher mortality rates.^9^ Similarly, using our TNM staging system, we observed that median overall survival was associated with disease stage, as higher stage disease corresponded with worse overall survival (Tables 3 and 4). A reliable, validated staging system is necessary as proper staging of UrC will aid in treatment decision‐making and may ultimately affect patient prognosis and survival outcomes.

Currently, no standard treatment guidelines exist for UrC but rather are extrapolated from small‐series case reports and single‐institution studies. According to The National Comprehensive Cancer Network (NCCN) guideline recommendations, patients with localized UrC should undergo partial or complete cystectomy with en bloc resection of the urachal ligament, umbilicus, and lymph node dissection.^20^ In a study by Siefker‐Radtke et al., cystectomy with en bloc resection of the urachal ligament and umbilectomy with negative margins led to improved long‐term survival. Surprisingly, their study found that complete versus partial cystectomy had no effect on survival.^21^ Multiple other studies found that partial cystectomy with en bloc removal of the urachal ligament and umbilicus was associated with improved survival.^7^, ^10^, ^22^, ^23^ However, a complete cystectomy should be considered if negative bladder surgical margins are unable to be obtained with a partial cystectomy as positive margins are associated with higher relapse rates.^8^, ^24^ Furthermore, pelvic lymph nodes are a common site of metastasis and disease relapse; however, the effectiveness of pelvic lymph node dissection remains controversial.^1^ Studies have shown nodal involvement to be a negative prognostic factor and associated with higher relapse rates and decreased survival.^21^, ^24^ Several studies recommend lymph node sampling to allow for more accurate staging of disease.^7^, ^10^ Regarding systemic therapy, NCCN recommends chemotherapy for node‐positive or advanced disease with leucovorin, 5‐fluorouracil (5‐FU), and oxaliplatin (FOLFOX) or Gemcitabine, 5‐FU, leucovorin, and cisplatin (GemFLP).^20^ According to Siefker‐Radtke et al., patients with metastatic disease responded better to GemFLP than 5‐FU, alpha‐interferon, and cisplatin or methotrexate, vinblastine, doxorubicin, cisplatin (M‐VAC).^21^ Likewise, Szarvas et al. showed that patients receiving 5‐FU with cisplatin had improved response rates and lower rate of progression when compared to cisplatin‐based treatments.^7^ Interestingly, there appears to be clinical and immunohistochemical similarities between UrC and colonic adenocarcinoma; thus it is thought that regimens such as FOLFOX, which are commonly used in gastrointestinal cancer, may be useful in relapsed UrC.^21^, ^24^ There remains no established role for neoadjuvant/adjuvant chemotherapy. Some authors believe neoadjuvant chemotherapy may play a role in node‐positive, unresectable disease that responds to systemic therapy and then requires surgical consolidation. Consideration for chemotherapy in the adjuvant setting should be given to patients who have an increased chance of relapse due to positive surgical margins, lymph node or peritoneal involvement, or did not undergo umbilectomy during en bloc resection.^20^, ^24^

An optimal staging system is necessary to help direct treatment algorithm. For the proposed TNM staging system, patients with stage 1, 2, and 3 disease may likely benefit from upfront surgical umbilectomy and partial or radical cystectomy, while stage 2 and 3, especially the latter may potentially benefit from adjuvant systemic therapy. Stage 4 disease would likely require upfront systemic chemotherapy and potentially salvage/consolidative surgical resection for those who responded to chemotherapy. Ongoing effort is being made to link the treatment modalities and disease stage.

Analyses utilizing the NCDB database have several limitations, so our results should be interpreted with caution. For instance, the AJCC TNM staging system for the urinary bladder cannot be directly applied to UrC; however, per the NCDB PUF, UrC cases were coded according to the 6th and 7th editions of the AJCC Cancer Staging Manual for the urinary bladder.^14^, ^15^ Furthermore, the 7th edition of the AJCC Cancer Staging Manual for the urinary bladder had changes in staging, grading, and nodal classification when compared to the 6th edition.^15^ Although some studies have shown the applicability of the TNM staging system for UrC,^9^, ^10^ there remain inherent limitations in using the urinary bladder TNM staging system as UrC does not develop from the bladder surface urothelium and exhibits clinical and pathologic characteristics distinct from bladder cancers.^9^ Additionally, while the NCDB PUF stipulates that case coding should be completed by an attending physician, registry staff were frequently involved in the coding process by reviewing clinical and patient notes provided to them. Due to the strict diagnostic criteria associated with UrC, it is critical that patient records are reviewed by a trained physician with a thorough understanding of the disease to minimize human error during the case‐coding process. Unfortunately, the NCDB does not disclose whether the cases were coded after careful review by a pathologist. Additionally, there exist selection biases within our analysis. While the NCDB contains information on a large number of newly diagnosed cancers in the United States, it does not represent the true population. This is primarily due to fact that only CoC‐designated hospitals disclose data to the NCDB, which means that patients who received care at non‐CoC hospitals are not included in the database.^25^ Due to the inherent limitations of our analysis, our findings should not be regarded as conclusive but instead as hypothesis generating.

## 5 CONCLUSION

UrC is an extremely rare malignancy with advanced stage on presentation that lacks a validated staging system or definitive treatment guidelines, largely in part due to the lack of large, randomized, prospective studies. We describe here a novel, four‐category TNM staging system constructed from a large population‐based analysis. We hope that our proposed staging system allows for improved risk‐stratification, prognostication, and therapeutic decision‐making. Future studies will aim at performing a retrospective review of overall survival, relative to treatment modalities, in UrC.

## AUTHORS CONTRIBUTION

Vladimir Limonnik: Conceptualization, methodology, formal analysis, writing‐ original draft, writing‐ review and editing. Arash Samiei: Methodology, formal analysis, writing‐ review and editing. Stephen Abel: Methodology, formal analysis, writing‐ review and editing. Rodney E. Wegner: Methodology, formal analysis, writing‐ review and editing. Goutham Vemana: Formal analysis, writing‐ review and editing. Shifeng S. Mao: Conceptualization, methodology, formal analysis, writing‐ review and editing, supervision, project administration.

## FUNDING INFORMATION

There was no funding for this study.

## CONFLICT OF INTEREST

All authors declare there are no conflicts of interest.

## ETHICAL STATEMENT

A retrospective review was performed using de‐identified patient data; given patient de‐identification, the study was exempt from institutional review board oversight.

## Data Availability

Not Applicable.

## References

[1] Paschke L , Juszczak M , Slupski M . Surgical treatment of recurrent urachal carcinoma with liver metastasis: a case report and literature review. World J Surg Oncol. 2016;296:1‐6. doi:10.1186/s12957-016-1057-4 PMC5126806 27894318

[2] Gopalan A , Sharp D , Fine S , et al. Urachal carcinoma: a clinicopathologic analysis of 24 cases with outcome correlation. Am J Surg Pathol. 2009;33(5):659‐668. doi:10.1097/PAS.0b013e31819aa4ae 19252435 PMC4225778

[3] Grignon DJ , Ro JY , Ayala AG , Johnson DE , Ordóñez NG . Primary adenocarcinoma of the urinary bladder. A clinicopathologic analysis of 72 cases. Cancer. 1991;67(8):2165‐2172. doi:10.1002/1097-0142(19910415)67:8<2165::aid-cncr2820670827>3.0.co;2-m 1706216

[4] Sheldon CA , Clayman RV , Gonzalez R , Williams RD , Fraley EE . Malignant urachal lesions. J Urol. 1984;131(1):1‐8. doi:10.1016/s0022-5347(17)50167-6 6361280

[5] Reis H , Szarvas T . Urachal cancer‐current concepts of a rare cancer. Pathologe. 2019;40:31‐39. doi:10.1007/s00292-018-0516-9 30895340

[6] Ashley R , Inman B , Sebo T , et al. Urachal carcinoma: clinicopathologic features and long‐term outcomes of an aggressive malignancy. Cancer. 2006;107(4):712‐720. doi:10.1002/cncr.22060 16826585

[7] Szarvas T , Modos O , Niedworok C , et al. Clinical, prognostic, and therapeutic aspects of urachal carcinoma‐ a comprehensive review with meta‐analysis of 1,010 cases. Urol Oncol. 2016;34(9):388‐398. doi:10.1016/j.urolonc.2016.04.012 27267737

[8] Hamilou Z , North S , Canil C , et al. Management of urachal cancer: a consensus statement by the Canadian Urological Association and genitourinary medical oncologists of Canada. Can Urol Assoc J. 2020;14(3):E57‐E64. doi:10.5489/cuaj.5946 31348743 PMC7053367

[9] Dhillon J , Liang Y , Kamat A , et al. Urachal carcinoma: a pathologic and clinical study of 46 cases. Hum Pathol. 2015;46(12):1808‐1814. doi:10.1016/j.humpath.2015.07.021 26364859 PMC4824311

[10] Molina JR , Quevedo JF , Furth AF , Richardson RL , Zincke H , Burch PA . Predictors of survival from urachal cancer: a Mayo Clinic study of 49 cases. Cancer. 2007;110(11):2434‐2040. doi:10.1002/cncr.23070 17932892

[11] Pinthus J , Haddad R , Trachtenberg J , et al. Population based survival data on urachal tumors. J Urol. 2006;175(6):2042‐2047. doi:10.1016/S0022-5347(06)00263-1 16697798

[12] National Cancer Database . Accessed January 15, 2020. https://www.facs.org/quality‐programs/cancer/ncdb

[13] Amin M , Edge S , Greene F , et al.; American Joint Committee on Cancer . AJCC Cancer Staging Manual. 8th ed. Springer; 2018. Accessed January 18, 2020. https://link.springer.com/book/9783319406176

[14] Greene F , Page D , Fleming I , et al.; American Joint Committee on Cancer . AJCC Cancer Staging Manual. 6th edition, Springer; 2002. Accessed January 19, 2020. https://cancerstaging.org/referencestools/deskreferences/documents/ajcc6thedcancerstagingmanualpart1.pdf

[15] Edge S , Byrd D , Compton C , et al.; American Joint Committee on Cancer. AJCC Cancer Staging Manual. 7th ed., Springer; 2010. Accessed January 22, 2020. https://www.springer.com/us/book/9780387884424

[16] Singh R , Mukhopadhyay K . Survival analysis in clinical trials: basics and must know areas. Perspect Clin Res. 2011;2(4):145‐148. doi:10.4103/2229-3485.86872 22145125 PMC3227332

[17] Deyo RA , Cherkin DC , Ciol MA , Adapting a clinical comorbidity index for use with ICD‐9‐CM administrative databases, J Clin Epidemiol 45 (6) (1992) 613–619. doi: 10.1016/0895-4356(92)90133-8. Accessed February 23, 2020. http://www.ncbi.nlm.nih.gov/pubmed/1607900 1607900

[18] Wright JL , Porter MP , Li CI , Lange PH , Lin DW . Differences in survival among patients with urachal and nonurachal adenocarcinomas of the bladder. Cancer. 2006;107(4):721‐728. doi:10.1002/cncr.22059 16826584

[19] Bruins HM , Visser O , Ploeg M , Hulsbergen‐van de Kaa CA , Kiemeney LA , Witjes JA . The clinical epidemiology of urachal carcinoma: results of a large, population based study. J Urol. 2012;188(4):1102‐1110. doi:10.1016/j.juro.2012.06.020 22901574

[20] Flaig T , Spiess P , Agarwal N , et al., National Comprehensive Cancer Network Clinical Practice Guidelines in oncology version 5.2021. Bladder Cancer. Accessed April 20, 2021. https://www.nccn.org/professionals/physician_gls/pdf/bladder.pdf

[21] Siefker‐Radtke A , Gee J , Shen Y , et al. Multimodality management of urachal carcinoma: the M.D. Anderson Cancer Center experience. J Urol. 2003;169(4):1295‐1298. doi:10.1097/01.ju.0000054646.49381.01 12629346

[22] Henly D , Farrow G , Zincke H . Urachal cancer: role of conservative surgery. Urology. 1993;42(6):635‐639. doi:10.1016/0090-4295(93)90526-g 8256396

[23] Herr HW , Bochner BH , Sharp D , Dalbagni G , Reuter VE . Urachal carcinoma:contemporary surgical outcomes. J Urol. 2007;178(1):74‐78. doi:10.1016/j.juro.2007.03.022 17499279

[24] Seifker‐Radtke A . Urachal adenocarcinoma: a clinician's guide for treatment. Semin Oncol. 2012;39(5):619‐624. doi:10.1053/j.seminoncol.2012.08.011 23040259

[25] Palma DA . National Cancer Data Base: an important research tool, but not population‐based. J Clin Oncol. 2017;35(5):571. doi:10.1200/JCO.2016.69.2855 27870569

